# Infective Diseases

**Published:** 1895-05-25

**Authors:** 


### Progress in Medicine.

### INFECTIVE DISEASES.

**Malaria.**—A flood of light from various sources is being poured on the ætiology of malarial fevers. The recently translated monographs of Mannaberg and Marchiafava and Bignami, enable us to classify these diseases into two groups, the mild (quartans and most tertians) and severe type (quotidian and some tertians). The latter forms alone are characterised by the presence of parasites resembling crescents, and forming spores.[1] Spontaneous cure may take place through the phagocytic action of the large cells of the spleen and marrow, the destructive action of the fever, and the sterility of many of the parasites. The action of quinine is said by Mannaberg to be most energetic on the spores, and if administered a few hours before the febrile attack, they are killed as they are formed. Crescents are conjugated individuals, and on them quinine has no effect. However, the question of the specific difference of the various clinical types is still one on which many observers differ, but the presence of these protozoa under one form or other in the blood of malarial patients has been shown in most parts of the world.[2] Osler reports on them as found in America. He divides them into the mild types, tertian and quartan,[3] in which, however, double infection may produce quotidian and other clinical forms; and the severe type, more often irregular in its paroxysms, presenting the crescent bodies, and occurring later in the year. His colleagues do not support the view that crescents are the result of conjugation. P. Manson, in the Hunterian Oration, agreed with this classification[4], and with the statement of Osler, that in the severe type the rosette or sporulating, and the pigmented bodies are chiefly found in the spleen and other viscera, and but rarely in the circulation. It is estimated that a million and a half of our Indian fellow subjects are killed annually by these microbes. Among the symptoms produced by them are coma from cerebral thrombosis, anæmia, choleraic diarrhœa, and apoplectiform attacks, often referred to sunstroke. Hence the efficacy of injections of quinine in so-called heat apoplexy. A beautifully coloured chart of nearly 200 forms of the parasite is given by Manson in the *British Medical Journal* of December 1st, and will be found most useful for reference. His view of their life history[5] is that the crescentic and allied forms are intended to pass outside the human body through the agency of some suctorial insect, such as the mosquito, and that the flagella themselves are a form proper to the new habitat. At the Indian Congress, Crombie, as president in the section of Medicine and Pathology, confirmed the Italian discoveries by his microscopical observations, and remarked that by far the most common type in that country was the quotidian in which the rosette forms disappear from the circulation into the spleen and viscera. There are also found remittent malarial and non-malarial fevers. The former are amenable to quinine, and often present two or more broods of parasites whose development is not synchronous. In these, therefore, there is no period of apyrexia. The non-malarial types include (*a*) typhoid; (*b*) "14 day" or Calcutta fever, which lacks all the specific marks of typhoid, though resembling it superficially; (*c*) a persistent low fever with few symptoms or complications, cured by change of air; (*d*) a malignant fever of high temperature, hepatic enlargement, coma, and bilious diarrhœa, lasting about six weeks; and (*e*) simple continued fever, lasting one week or less.[6] The distinction of these types from each other, and especially from typhoid, is warmly discussed there and in America, and though Crombie most carefully enumerates the clinical distinctions, he does not report any thorough search for the typhoid bacillus. The diazo reaction is said by Atkinson, of Hong Kong, to be useful in distinguishing typhoid from some of the others.[7] Hughes, of Malta, seems to identify it (*d*) with Malta fever, in which a micrococcus capable of producing the disease in monkeys replaces the typhoid bacillus; but his account of the symptoms differs considerably from that of Crombie.[8] W. G. Thompson found malarial organisms in three cases of typhoid which were carefully studied. The activity of the malaria did not commence till after the typhoid had run part of its course. He concludes that the two diseases may exist simultaneously without influencing each other or producing a hybrid.[9] Chassiotis, of Athens, describes minutely a type of fever apparently Crombie's (*a*) which differs from typhoid and malaria in the absence of ulceration of Peyer's patches, splenic enlargement and the specific microbes, and, on the other hand, in the constant presence of a diplococcus.[10] The paper is one of great value and interest; Manson's explanation of the transmission of malaria by mosquitoes, or other insects, is identical with that advanced by C. Findlay, of Havanna, for yellow fever, though the latter recognises that many of the symptoms such as hœmaturia are due to secondary infection by the bacillus coli and similar organisms.[11]

The American papers teem with discussions on the question whether malaria is a water-borne disease like cholera or no. There is much evidence that it may be communicated by water, but we find also instances where the upturning of malarial soils caused attacks, though the patients drank pure water and ate no contaminated food. Thus Dalrymple,[12] of Rochelle, N.Y., reports that his own child, fed on sterilised milk and water, was attacked under these circumstances. The prophylactic value of arsenic, quinine, cinchona, and other drugs was reviewed by Duncan at the Indian Congress, and their effects shown to vary widely in different places, thus arsenic is valuable in Italy and the Congo, and has little effect in India.[13] There is a general agreement that overdoses of quinine as a preventative have an injurious effect on the health, and that the use of quinine in non-malarial remittents is most harmful. Still, quinine retains the first rank among the therapeutics of true malaria, though methyline blue, arsenic, and splenic extract are praised by various writers in different cases. Forbes, indeed, lays down that no single drug should be regarded as a specific in every case.

A *bruit de galop* over the tricuspid area is mentioned as a useful diagnostic sign in hypertrophy of the liver of malarial origin by C. Naamé. It disappears in a few days under the influence of quinine.[14] Though there is some evidence of an antagonism between malaria and phthisis, a pseudo-tuberculosis in chronic malarial subjects is, according to C. Duba,[15] shown by apical consolidation, with cough and irregular temperatures. Sometimes hœmoptysis and the absence of bacilli are noticed.

**Cerebro Spinal Meningitis.**—An exhaustive report on an outbreak in Maryland by Flexner and Barker, with an account of the bibliography of the subject, will be of interest with reference to the recent discussion on the pneumococcus infections.[16] Apart from cases secondary to ear and nose diseases, the mode of invasion of the pneumococcus is unknown. Some facts point to its entrance through the epithelium of the intestinal tract, but there is at present no clue to the cause which renders a large number of persons in a given locality vulnerable. Ferguson also found the pneumoccus in sections of the brain and spinal cord in the majority of cases he had examined. Isolated cases are not infrequently met with in America and occasionally in this country, and may occur in connection with pneumonia and other diseases. The symptoms of some fourteen cases occurring in St. Bartholomew's Hospital are analysed by Omerod. In the later ones the pneumococcus was sought for and found. Cases secondary to other diseases are excluded, but no new light is thrown on its production.

**Cholera.**—The influence of erysipelas on this disease is discussed by Blagoveshtchensky,[17] who mentions four consecutive patients who were attacked by erysipelas under the influence of which the disease rapidly changed its type and all recovered. Poehl[18] records the results of spermin in cholera, and offers a supply of the remedy free for further trials. Freymuth injected the blood serum of convalescent cholera patients in doses of 10 to 50 c.c. daily, with apparent success in a few cases.[19] At the Indian Congress many papers of interest were read on the disease. Herbert holds that it is endemic in the greater part of India and not merely in the Ganges delta. Rainfall, temperature, and atmospheric pressure have all some connection with outbreaks, but mainly through their influence on the water supply.[20] Where the ground water level is very low indeed, there is little or no cholera; where it is moderately low, cholera comes with the rains; where it is very high, cholera exists all the year, but decreases during the flood time. Lewtas[21] argued that epidemics are not spread faster by railways than by slower means of travel, and do not follow the line of railway traffic in preference to other routes. Haffkine[22] gave a summary of the results of his inoculations in 32,146 persons, but a long while elaped before any were made where cholera actually existed. In Calcutta and Cawnpore very successful results followed; in Lucknow, the inoculated were attacked in a less degree only than the uninoculated. The comparative failure here might be due to the weakness of the vaccine or the lapse of time after vaccination; at all events, the harmlessness of the inoculation was established. The Health Officer of Calcutta, in his report, considers their temporary protective power fully proved, and that the partial failures may be obviated in future as better supplies of vaccine are forthcoming. In his opening address, Ernest Hart[23] carefully guarded himself from supporting the idea that "water-carriage covers the whole pathology of the disease or explains the ætiology of its occasional epidemic prevalence." Two lines of inquiry seemed to him to need especial investigation, first, the relation between the preceding or concurrent diarrhœa and the actual cholera, and secondly, the other organisms besides Koch's which may be engaged in the production of the disease. Hankin and his colleagues found Koch's bacillus in the well-water in nearly every place where an epidemic was going on. It was here shown to be in a virulent condition. Later on it would be still found, but with signs of degeneration and no longer fatal to guinea pigs. After three months, and in places where no cholera had existed, vibrios were very rarely met with. They found virulent bacilli actually in the meat and milk of a regimental cookhouse in Cawnpore, where it had been conveyed by the servants from the infected city, though the water supply of the troops was not infected.[24] Haffkine and Simpson gave confirmatory evidence from the water f tanks near Calcutta, and had found the microbe in the fæces of cows suffering from diarrhœa and in the water they were drinking. Hankin considers that potassium permanganate has almost a specific action on the bacilli, causing them to vanish from the water of wells where they are present. The Mahommedan community and the Nizam's Ministers of State at Hyderabad gave an enthusiastic reception to Ernest Hart on the question of the prevention of cholera in the Mecca pilgrimage.[25] He showed that cholera disappeared in India, wherever a pure water supply and the absence of contamination was provided for. Three hundred thousand pilgrims went annually from India, and owing to the miserable quarantine and sanitary arrangements a frightful spread of cholera was constantly taking place. Strongly worded resolutions were agreed to by the meeting praying for a reform of these matters. Abel and Claussen conclude from their investigations at Königsberg that cholera vibrios die in fæcal matter usually within twenty days, and rarely remain much longer.[26] They sometimes disappear in two or three days, and may exist in healthy persons who are exposed to infection. In such cases quarantine should be kept up till repeated examinations show the absence of the germs. Kolle[27] found, however, that bacilli were present in the stools of convalescents for periods of ten to forty-eight days. Pfeiffer[28] shows that the serum of immunised animals has bactericidal and protective powers in animals against the cholera virus when it is injected into the peritoneum. It does not neutralise the virus chemically, for the latter is unaffected if mixed with it in a glass, nor does it cause phagocytosis, but it appears to cause the production by the cells of defensive substances, just as occurs in diphtheria and tetanus. The experiments of W. Hesse show that fresh unboiled cow's milk is no culture medium for the bacilli. They may be conveyed in it for a short time only, but die almost without exception in twelve hours.

Metchnikoff believes that the immunity of human beings and other animals depends largely on the action on the cholera microbes of the other organisms of the digestive canal. There is also much evidence to show that the true bacilli often undergo change of form and characters. This has been noted in many Russian cases, but Terni and Pellegrini consider that though variation of form takes place there is no evidence for supposing that there is more than one species of vibrio which causes cholera.[29] Max Grüber regards Koch's bacillus as indistinguishable from others in some cases.[30] Koch's bacillus is undoubtedly present in most cholera outbreaks, but possibly indigenous vibrios are responsible for others. Even in typical cases some unknown factor as well as the bacillus is needed to account for the receptivity of the patient. Chantemesse shows that the bacillus of the mild Lisbon outbreak, which differed markedly from the true species, could actually be caused to revert to the Indian type by passing it through the bodies of animals, when it regained the indol reaction and other properties. It is therefore merely an acclimatised and degenerative type, derived from Koch's bacillus, and not from indigenous vibrios. Rumpf, of Hamburg, in a long review of the epidemic, insists upon the Indian origin of the germs[31] which, however, undergo long periods of development outside the human body, and change in virulence and activity. In Europe these changes are usually in a degenerative direction, but under favourable conditions they may regain some of their original power. We have yet to learn how long it is ere transplanted colonies finally die in these climates. In an epidemic, severe cases of cholera may develop in 50 per cent. of the infected, the numbers attacked varying, like those of indigenous diseases, with the prevalence of various conditions of ill-health in the infected, such as intestinal catarrh. It would be interesting to learn whether the various types so long cultivated by Cunningham from cholera cases in India can be transformed by any means to the type of Koch's bacillus.

[1] Dublin Med. J., Feb., 1895. [2] Lanc., Nov. 24, 1894. [3] B. M. J., Jan. 5, 1895. [4] Lanc., Feb. 16, 1895. [5] B. M. J., Dec. 8, 1895. [6] Lanc., Jan. 19, 1895. [7] Lanc., April 28, 1894. [8] Lanc., March 2, 1895. [9] Int. Med. Mag., Oct., 1894. [10] Fortsch. der Med., Nov. 15, 1894. [11] Ed. Med. J., Sept. and Nov., 1894. [12] N. Y. Med. Rec., Nov. 10, 1894. [13] Lanc., Jan. 19, 1895. [14] Med. Week, Oct. 13, 1894. [15] B. M. J., Dec. 29, 1894. [16] Am. J. Med. Sc , March, 1894. [17] B. M. J., Oct. 6, 1894. [18] B. M. J., Oct. 6, 1894. [19] Deut. Med. Woch., p. 43, 1894. [20] Lanc., Jan. 12, 1895. [21] Jan. 19, 1895. [22] B. M. J., Jan. 26, 1895. [23] B M. J., Dec. 29, 1894, [24] B. M. J., Feb. 9, 1895. [25] B. M. J., March 16, 1895. [26] Lanc., Feb. 23, 1895. [27] Med. Week., Dec. 28, 1894. [28] Fortsch. der Med., Dec. 15, 1894. [29] N. Y. Med. J., Dec. 1, 1894. [30] Med. Week., Sept. 21. [31] Klin. Vortrage, No. 109-10.

